# A Myasthenic Crisis Complicated by a Takotsubo Cardiomyopathy

**DOI:** 10.7759/cureus.21067

**Published:** 2022-01-10

**Authors:** Amit Ramrattan, Iovank Gonzalez, Harun Abdullah, Kevin Maraj, Mariana Browne

**Affiliations:** 1 Internal Medicine, Port of Spain General Hospital, Port of Spain, TTO; 2 Internal Medicine/Cardiology, Port of Spain General Hospital, Port of Spain, TTO; 3 Integrative Medicine, Port of Spain General Hospital, Port of Spain, TTO

**Keywords:** echo cardiogram, stress-related cardiomyopathy, myasthenic crisis, takotsubo cardioyopathy, myasthenia gravis (mg)

## Abstract

Myasthenia gravis (MG) is an autoimmune disorder of the neuromuscular junction that affects skeletal muscles causing weakness, typically the ocular, facial, oropharyngeal, respiratory, and limb muscles. Patients can present as either an MG exacerbation with weakness of any muscle group or an MG crisis which is a life-threatening weakness of the respiratory muscles that usually requires intubation and mechanical ventilation; however, though rare, cardiac manifestations must be considered in the management of such patients.

## Introduction

Takotsubo cardiomyopathy (TCM) has become a distinct clinical entity over the past 20 years, diagnosed by the modified Mayo Clinic criteria [1]. It is characterized by a sudden and transient wall motion abnormality of the apex of the left ventricle (LV), resembling the Japanese octopus fishing pot (takotsubo), usually precipitated by emotional or physical stress and carries a good prognosis, with up to 95% making a full recovery in four to eight weeks [2]. Though a rare occurrence, an myasthenia gravis (MG) crisis can be a precipitant to the development of a TCM. This is the case report of a patient who presented to the Port of Spain General Hospital with a myasthenic crisis and subsequently developed a TCM as a complication of this event, which is the first case reported in the Caribbean.

## Case presentation

A 70-year-old female patient presented to the Port of Spain General Hospital with a one-day history of dyspnea. Six years prior, she was diagnosed with thymoma-positive myasthenia gravis after which she had a thymectomy complicated with a left phrenic nerve palsy post-op followed by radiation therapy, after which she was maintained with pyridostigmine and low dose prednisolone alternating 5/7.5 mg daily. Some months prior to this current admission, she was managed at another tertiary institution for a non-ST elevation myocardial infarction (NSTEMI). Records from this prior admission could not have been attained as this was in another country. However, the patient never underwent cardiac catheterization during that time.

**Figure 1:**
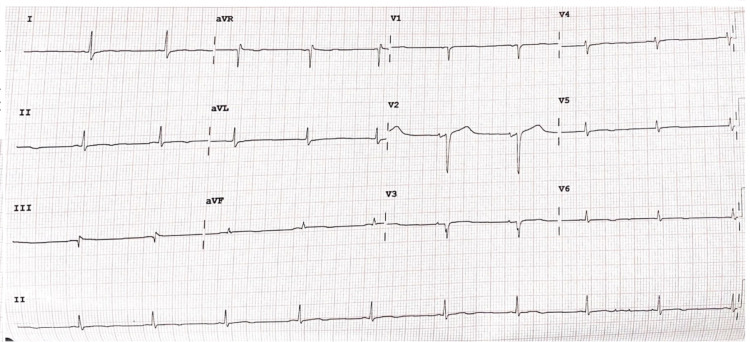
Electrocardiogram on admission shows sinus rhythm with a rate of 60 beats per minute aVR: augmented vector right; aVL: augmented vector left; aVF: augmented vector foot

**Figure 2:**
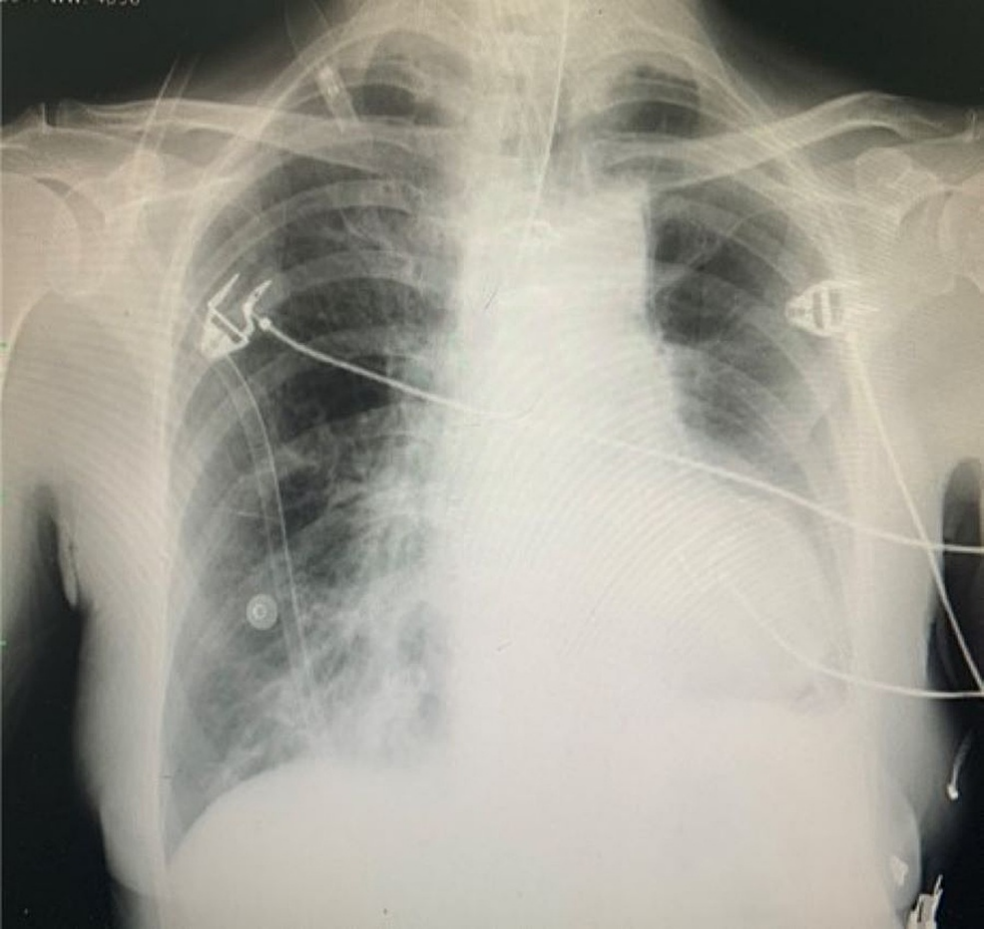
Post-intubation chest x-ray

On this admission, this patient had a type 2 respiratory failure with pH 7.23, partial pressure of carbon dioxide (pCO_2_) 54 mmHg, partial pressure of oxygen (pO_2_) 45 mmHg, and HCO_3_ 22.3 mEq/L. Blood pressure (BP) was 99/59 mmHg with a pulse of 99. Complete blood count showed a white cell count of 10.3 x 10^9^/L, hemoglobin (Hb) of 10.3 g/L, platelet of 200 g/L with normal partial thromboplastin time/prothrombin time (PT/PTT), and renal function test. Her admission ECG and post-intubation chest x-ray (CXR) are shown in Figure 1 and Figure 2.

Admission ECG showed a sinus rhythm with poor R-wave progression and a right axis deviation and CXR with right-sided consolidation. Given her right pneumonia and already type 2 respiratory failure, this patient was assessed as a myasthenic crisis (MC) and was intubated and admitted to the intensive care unit. She was commenced on pyridostigmine 60 mg PO TDS, hydrocortisone 100 mg IV TDS, IVIg 20g daily for five days, ceftriaxone 1g IV OD and DVT prophylaxis with enoxaparin 40 mg SC OD along with acetylsalicylic acid (ASA) 81 mg daily from prior.

**Figure 3:**
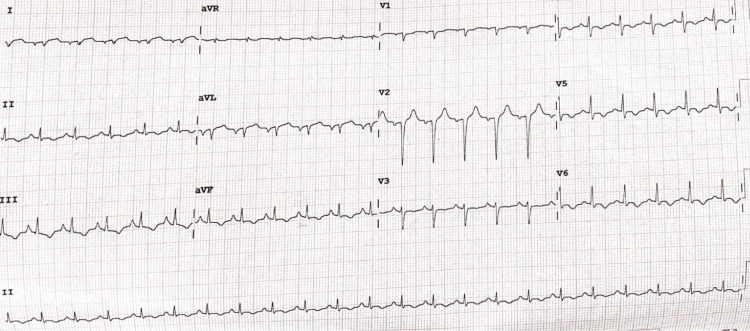
Day 3 electrocardiogram shows sinus tachycardia with a rate of 136 bpm in addition to T-wave inversions in leads 2, 3, aVF, V4, V5 and V6 aVR: augmented vector right; aVL: augmented vector left; aVF: augmented vector foot

**Figure 4:**
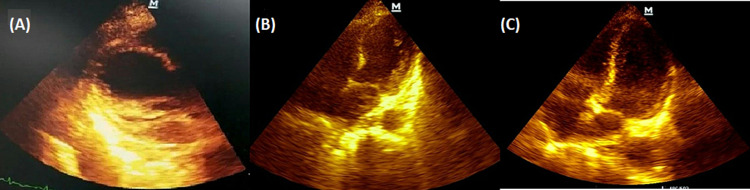
Echocardiogram showing (A) apical ballooning, parasternal long-axis view, and (B) improvement of apical ballooning, apical four-chamber view. (C) Echocardiogram of day 14, apical four-chamber view

On day three of admission, the patient started experiencing palpitations after which an ECG was done showing new lateral wall T-wave inversions and was then found to have an elevated troponin T-HS of 475 pg/ml (Figure 3). On day four of admission, she was commenced on a dopamine infusion due to cardiogenic shock with a BP of 80/51 mmHg after which her pyridostigmine was withheld as this was suspected to be a contributing factor to the patient's hypotensive state or a rare precipitator of a cholinergic crisis picture. An echocardiogram was done showing apical ballooning with ejection fraction 30-35% in keeping with a TCM (Figure 4A). Three days after, another echocardiogram was done which showed less apical ballooning with moderated left ventricular (LV) dysfunction (Figure 4B) and by day 14 of admission, her echocardiogram showed normal LV function and no regional wall motion abnormalities and a new EF of 65% (Figure 4C). During this time period, the patient's respiratory status had improved, was extubated, and subsequently discharged having been reestablished on pyridostigmine.

## Discussion

The exact pathophysiology of TCM is unknown but there is supporting evidence, given the emotional and physical stressors, that a catecholamine surge drives this process [3]. Within the apex of the myocardium is a high density of beta 2-adrenergic receptors and during a catecholamine surge, there is a switch in the G-protein signaling pathway from stimulatory to inhibitory, a term known as ligand-directed trafficking. It is this inhibition that accounts for the regional hypokinesia seen in the apex of the left ventricle in a TCM as well as the quick resolution of the disease process as catecholamine levels normalize. This neurogenic or catecholamine stunning, though impairing cardiac contractility, is suspected to be cardioprotective against the effects of this neurotransmitter, thus accounting for only a modest elevation of troponin levels [4]. This catecholamine surge is theorized to occur in cases of emotional or physical stress leading to the development of a TCM.

Our patient, however, had a myasthenic crisis and there have been case reports published showing a link between MG and TCM. In a systematic review of all documented cases of TCM with MG [5], 88% of cases were found in the Western Pacific, American, and European regions with 81% females affected, thus mirroring that TCM is a disease of mainly post-menopausal women [6]. This retrospective case review further observed that in more than half the cases, there were left systolic dysfunction with elevated troponin levels and T-wave inversions.

One important differential diagnosis to take into consideration in an MG patient in cardiogenic shock is myocarditis as 48% of all MG cases and 97% of all thymoma-associated MG have antibodies towards heart muscle [7]. Given the echocardiogram findings, in this case, myocarditis is less likely as in this condition, one would expect there to be global hypokinesia instead of specific regional wall motion abnormality like the apical ballooning noted in our patient.

TCM was the likely diagnosis in this case when using the International Takotsubo Diagnostic Criteria (InterTAK diagnostic criteria) as opposed to the Mayo Clinic criteria (Tables 1, 2) [8,9]. This post-menopausal patient exhibited echocardiographic features after a stressor event presenting in a myasthenic crisis along with ECG changes and elevations in cardiac biomarkers. What was lacking in this case was the availability of services for cardiac MRI, which in TCM, shows an absence of delayed gadolinium hyperenhancement [10-12]. This differs from myocardial infarction and myocarditis where the opposite occurs. Cardiac MRI can also assess for regional wall motion abnormalities in both right and left ventricles as well as apical left ventricular thrombi.

**Table 1:** International Takotsubo Diagnostic Criteria (InterTAK Diagnostic Criteria)

International Takotsubo Diagnostic Criteria (InterTAK Diagnostic Criteria)
1. Patients show transient left ventricular dysfunction (hypokinesia, akinesia, or dyskinesia) presenting as apical ballooning or midventricular, basal, or focal wall motion abnormalities.
2. An emotional, physical, or combined trigger can precede the takotsubo syndrome event, but this is not obligatory
3. Neurologic disorders (e.g., subarachnoid hemorrhage, stroke/transient ischaemic attack, or seizures), as well as pheochromocytoma, may serve as triggers for takotsubo syndrome
4. New ECG abnormalities are present (ST-segment elevation, ST-segment depression, T-wave inversion, and QTc prolongation); however, rare cases exist without any ECG changes
5. Levels of cardiac biomarkers (troponin and creatine kinase) are moderately elevated in most cases; significant elevation of brain natriuretic peptide is common
6. Significant coronary artery disease is not a contradiction in takotsubo syndrome
7. Patients have no evidence of infectious myocarditis
8. Postmenopausal women are predominantly affected

**Table 2:** Modified Mayo Clinic criteria

Modified Mayo Clinic criteria
1. Transient hypokinesis, dyskinesis, or akinesis of the LV midsegments, with or without apical involvement
2. Absence of obstructive coronary disease or angiographic evidence of acute plaque rupture
3. New ECG abnormalities or elevation in the cardiac troponin level
4. Absence of significant head injury/ICH, pheochromocytoma or myocarditis, and hypertrophic cardiomyopathy

As mentioned in the case history, this patient suffered an NSTEMI prior to this presentation. Whether this is a recurrence of a TCM remains unknown as the details of that prior hospitalization could not be obtained as it was in another country. This clinical vignette has been documented in a case report by Battineni et al. which may have further supported the diagnosis of this case presentation [13].

## Conclusions

When a patient presents with features of a myasthenic crisis, clinicians must be vigilant for the possible development of cardiac complications such as TCM and myocarditis, as dyspnea from a cardiac cause can be easily overlooked. We recommend that once a patient presents with a crisis that close cardiac monitoring is done with ECG and serial troponins and if abnormal, an echocardiogram be arranged. Though a limiting factor in this case, an angiogram should be done to exclude concomitant coronary artery disease and if thymoma positive, a cardiac MRI to exclude myocarditis.
